# Ion-Channel Genosensor for the Detection of Specific DNA Sequences Derived from Plum Pox Virus in Plant Extracts

**DOI:** 10.3390/s141018611

**Published:** 2014-10-09

**Authors:** Kamila Malecka, Lech Michalczuk, Hanna Radecka, Jerzy Radecki

**Affiliations:** 1 Institute of Animal Reproduction and Food Research, Polish Academy of Sciences, Tuwima 10, 10-748 Olsztyn, Poland; E-Mails: k.malecka@pan.olsztyn.pl (K.M.); h.radecka@pan.olsztyn.pl (H.R.); 2 Research Institute of Horticulture, Konstytucji 3 Maja 1/3, 96-100 Skierniewice, Poland; E-Mail: lech.michalczuk@inhort.pl

**Keywords:** ion-channel sensor, DNA biosensor, square wave voltammetry, Plum Pox Virus, plant extracts, glassy carbon electrode

## Abstract

A DNA biosensor for detection of specific oligonucleotides sequences of Plum Pox Virus (PPV) in plant extracts and buffer is proposed. The working principles of a genosensor are based on the ion-channel mechanism. The NH_2_-ssDNA probe was deposited onto a glassy carbon electrode surface to form an amide bond between the carboxyl group of oxidized electrode surface and amino group from ssDNA probe. The analytical signals generated as a result of hybridization were registered in Osteryoung square wave voltammetry in the presence of [Fe(CN)_6_]^3−/4−^ as a redox marker. The 22-mer and 42-mer complementary ssDNA sequences derived from PPV and DNA samples from plants infected with PPV were used as targets. Similar detection limits of 2.4 pM (31.0 pg/mL) and 2.3 pM (29.5 pg/mL) in the concentration range 1–8 pM were observed in the presence of the 22-mer ssDNA and 42-mer complementary ssDNA sequences of PPV, respectively. The genosensor was capable of discriminating between samples consisting of extracts from healthy plants and leaf extracts from infected plants in the concentration range 10–50 pg/mL. The detection limit was 12.8 pg/mL. The genosensor displayed good selectivity and sensitivity. The 20-mer partially complementary DNA sequences with four complementary bases and DNA samples from healthy plants used as negative controls generated low signal.

## 1. Introduction

Plum Pox Virus, also known as Sharka (*Plum Pox*) is one of the most devastating viral diseases of stone fruits worldwide, with a significant impact on agronomy and economics. The disease is caused by the *Plum Pox Virus* (PPV), a member of the *Potyvirus* genus in the *Potyviridae* family. It not only damages plums, but also apricots, nectarines, sweet cherries and tart cherries. PPV is also able to infect important ornamental and wild *Prunus* L species, including those used in the traditional medicine: myrobalan, the American plum, and dwarf flowering almond and blackthorn. PPV is the most widespread disease of stone fruits in Europe. Symptoms of Sharka can be evident, as well as very subtle on stone fruit trees. This virus usually affects both the fruit and the leaves. The intensity of fruit symptoms is usually significantly enhanced by the age of the infected plant. This virus reduces fruit yield and quality. It also shortens the productive lifespan of the plantations and may cause stone fruit trees to become useless for fruit production. Even asymptomatic trees produce diminished quantities of fruit [1–3].

The conventional, most popularly applied techniques for PPV detection is enzyme-linked immunosorbent assay (ELISA) and polymerase chain reaction (PCR) [4–8]. Biosensors are a very promising alternative to the currently used traditional methods [9–12]. They have a simple construction and low cost. It is possible to miniaturize them. Thanks to biosensors, detection is simple and fast. The high sensitivity, compatibility with modern micro-fabrication technologies, inexpensive portability, and that they are label-free make them excellent candidates for a wide variety applications in areas such as medical diagnostics, forensics, biodefense, food contamination and environmental monitoring [13–15].

Nucleic acids are an extremely useful tool for this analytical application due to their powerful recognition properties [16,17]. In recent years, there has been a great interest in the use of nucleic acids as an instrument in biosensing [18,19]. A variety of techniques have been developed for the detection of DNA hybridization, including electrochemiluminescence [20,21], fluorescent [22,23], quartz crystal microbalance [24,25], surface plasmon resonance [26,27], piezoelectric [28–30] and electrochemical techniques [29,31–36]. The electrochemical DNA biosensors provide a novel technique for gene detection, and it will be of practical value in molecular biology and modern biomedical engineering owing to its simplicity and convenience in use compared to conventional probe labeling techniques [37–41].

Here, we report ion-channel electrochemical genosensor for the detection of specific DNA sequences derived from Plum Pox Virus in plant extracts and in buffer. For this purpose an NH_2_-ssDNA probe was attached to the glassy carbon electrode (GCE) surface through amide bonds. The signals generated as a result of hybridization reaction have been registered by Osteryoung square wave voltammetry (OSWV) in the presence of [Fe(CN)_6_]^3−/4−^ as a redox marker. The genosensor sensitivity and selectivity were tested with three types of targets, 22-mer, 42-mer ssDNA (single stranded DNA) sequences of oligonucleotides and DNA samples from extracts derived from healthy plants (C^−^ PPV) and plants infected with PPV (C^+^ PPV). As a negative controls 20-mer partially complementary (PC PPV) DNA sequences (4 complementary bases) and DNA samples from extracts derived from healthy plants (C^−^ PPV) were applied.

## 2. Experimental Section

### 2.1. Materials and Chemicals

2-morpholinoethanesulfonic acid (MES), *N*-hydroxysuccinimide (NHS), *N*-(3-dimethylaminopropyl)-*N*’-ethylcarbodiimide hydrochloride (EDC), ethanolamine, potassium ferro- and ferricyanides, phosphate PBS buffer components (NaCl, KCl, Na_2_HPO_4_, KH_2_PO_4_) were purchased from Sigma-Aldrich (Poznań, Poland). Alumina 0.3 and 0.05 μm was purchased from Buehler (Lake Bluff, IL, USA). Sulphuric acid, hydrogen peroxide and methanol were purchased from POCh (Gliwice, Poland).

Specific DNA sequences of Plum Pox Virus (PPV) were provided by the Research Institute of Horticulture in Skierniewice, Poland.

The modified oligonucleotide NH_2_-ssDNA (5′-NH_2_-(CH_2_)_6_-AGG GGA GTG TAG TGG TCTCGG T-3′) was used as a probe for immobilization on a glassy carbon electrode surface, while three unmodified oligonucleotides, 42-mer long complementary sequences (LC, 5′-*ACCGAGACCACTACACTCCCCTCA* CAC CGA GGA GGT TGT GCA-3′) and 22-mer short complementary sequences (SC, 5′-*ACCGAGACCACTACACTCCCCT*-3′) served as hybridization targets, respectively. A partially complementary sequence (PC, 5′-CTT *C*T*T* CTC TCT CCT *T*GA *G*G-3′) was used as a negative control. As target DNA, samples from extracts derived from plants infected with PPV (C^+^ PPV) were also applied, and as a negative control in this case DNA samples from extracts derived from healthy plants (C^−^ PPV) were used.

All aqueous solutions were prepared using Milli-Q water, resistivity 18.2 MΩ·cm (Millipore, Darmstadt, Germany). Reagents and solvents were of analytical grade and were used without further purification. Experiments were carried out at room temperature.

### 2.2. Preparation of DNA Samples from Extracts Derived from Healthy Plants and Infected with PPV

Fresh plant tissues needed for analysis were collected from the greenhouse (healthy material) and an experimental field (material infected with PPV). Sampled were tested for the presence of PPV virus by DAS-ELISA using polyclonal antibodies. Samples to isolate the genetic material of plants uninfected (C^−^ PPV) and infected with PPV (C^+^ PPV) were selected based on the results obtained from ELISA tests. The total RNA was extracted from plant tissue using the method described by Chang *et al.* [42]. Collected RNA preparations were digested with RQ-1 DNase (Promega, Mannheim, Germany), and then purified using the QIAGEN-RNeasy Plant Mini Kit. Then, RNA concentrations were measured spectrophotometrically using Epoch apparatus. The purified preparations of total RNA were translated in the reverse transcription reaction into cDNA using the RT enzyme M-MLV (Promega, Mannheim, Germany). Finally, DNA samples were purified using the QIAGEN-Qiaquick PCR Purification Kit and were used as targets C^+^ (infected with PPV) and C^−^ PPV (healthy material, negative control).

### 2.3. Preparation of PPV-Genosensor

Glassy carbon electrodes (GCE) (BioAnalytical System, BAS, West Lafayette, IN, USA), rinsed with methanol, were initially polished using microcloth polishing pad, with 0.3 and 0.05 μm alumina slurries (Alpha and Gamma Micropolish; Buehler, Lake Bluff, IL, USA) for 5 min each. After this step electrodes were carefully washed and sonicated in Milli-Q water for 1 min. The pre-treatment procedure to generate carboxylic groups on the electrode surface was performed in 0.5 M H_2_SO_4_ using conventional three-electrode electrochemical cell (GCE as a working electrode, Ag/AgCl reference electrode and Pt counter electrode). Measuring conditions were as follows: minimum potential −0.3 V, maximum potential 1.5 V, scan rate 100 mV/s, number of cycles 20. After finishing electrochemical pre-treatment electrodes were washed with Milli-Q water and placed in water (for several minutes, until the next step) to avoid contaminants from air. Afterwards, GCE were soaked in a mixture of 0.1 M EDC and 0.05 M NHS in 0.05 M MES pH 5.5, and their surface were activated for 1 h. Subsequently, electrodes were rinsed with 0.05 M MES, fixed upside down and 10 μL droplets of 10 μM NH_2_-ssDNA in 0.05 M MES buffer were spotted on each GCE surface for 3 hours. The residual NHS esters were blocked with 0.1 M ethanolamine pH 9.0 [43,44]. Finally, electrodes were rinsed with 0.05 M MES (pH 7) and 0.1 M PBS buffer pH 7.4 (0.137 M NaCl, 0.0027 M KCl, 0.01 M KH_2_PO_4_, 0.0018 M Na_2_HPO_4_), respectively. Fully modified electrodes were stored in 0.1 M PBS pH 7.4 until use, but no longer than one day.

### 2.4. Hybridization Processes

The CS, LC and PC targets were diluted in the hybridization buffer (0.1 M PBS, pH 7.4) to the concentration of 1, 2, 4, 6 and 8 [pM]. The DNA samples from healthy (C^−^ PPV) and infected plants (C^+^ PPV) were diluted in the hybridization buffer (0.1 M PBS, pH 7.4) to the concentration of 10, 30 and 50 [pg/mL]. Hybridization reactions were performed by dropping of the 10-μL aliquots of the respective dilutions of the targets on the NH_2_-ssDNA modified GCE surface. After 1 h of incubation at room temperature, the electrodes were rinsed with 5 mL of 0.1 M PBS, pH 7.4 in order to remove the unbound targets.

### 2.5. Cyclic Voltammetry (CV) and Osteryoung Square Wave Voltammetry (OSWV) Measurements

All electrochemical measurements were performed with a potentiostat-galvanostat AutoLab (Eco Chemie, Utrecht, The Netherlands) with a three-electrode configuration. Potentials were measured versus the Ag/AgCl electrode, and a Pt wire was used as the auxiliary electrode. The voltammetric experiments were carried out in an electrochemical cell of 5 mL volume. The measurements were performed in the presence of 0.1 M PBS buffer and 1.0 mM K_3_[Fe(CN)_6_]/K_4_[Fe(CN)_6_] (1:1) purged with nitrogen for 10 min, in order to control electrode modification and to record the hybridization processes.

OSWV was performed with potential from + 0.6 V to −0.1 V and with a step potential of 0.001 V, a square-wave frequency of 25 Hz and amplitude of 0.05 V for [Fe(CN)_6_]^3−/4−^. In CV, potentials were cycled from +0.6 V to −0.2 V with the scan rate of 0.1 V/s.

The electrode responses were expressed as: (I_n_ – I_0_)/I_0_ where I_n_ is the peak current measured in the presence of the target and I_0_ the peak current before applying the target (in buffer without the target).

## 3. Results and Discussion

### 3.1. Fabrication and Working Principle of Genosensor

The working principle of the genosensor proposed is based on an ion-channel mechanism originally developed by Umezawa [45]. In the case of the DNA biosensors, the active layer is a negatively charged ssDNA probe. Intermolecular recognition process responsible for generating the analytical signal is carried out by hybridization reaction between negatively charged probe disposed on the electrode surface and the negatively charged complementary sequence present in the sample solution. The negatively charged bioactive layer and negatively charged marker [Fe(CN)_6_]^3−/4−^, present in the solution, are repelled. The consequence of hybridization process increases the negative charge on the electrode surface, which hinders accessibility of negatively charged marker ions to the electrode surface. The larger negative charge causes stronger signal, and therefore the 42-mer sequence generates a stronger response—a greater decrease in the peak current compared to the 22-mer sequence.

The NH_2_-ssDNA probe has been covalently attached to the surface of glassy carbon electrodes through amide bond. The hybridization events with target ssDNA sequence were detected with [Fe(CN)_6_]^3−/4−^ redox marker present in the sample solution. The advantage of this approach is that oligonucleotides labeling is not necessary. Thus, such genosensor belongs to the ‘label free’ category.

**Figure 1.**
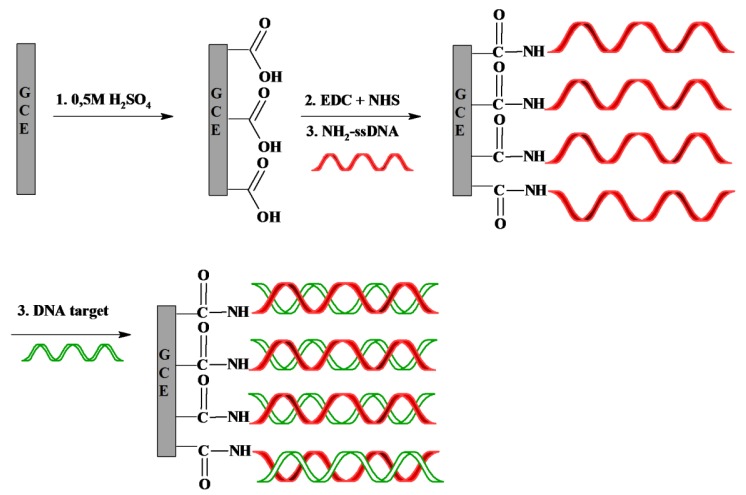
Schematic representation of the proposed genosensor.

The scheme of the genosensor fabrication is shown in Figure 1. First, carboxylic groups are created by oxidation of the GCE surfaces in 0.5 M H_2_SO_4_ [46]. Then, GCE were coated with NH_2_-ssDNA by covalent linkage to the carboxyl moiety on the electrode surface after activation by the mixture of EDC and NHS. The residual NHS esters were blocked with ethanolamine.

**Figure 2.**
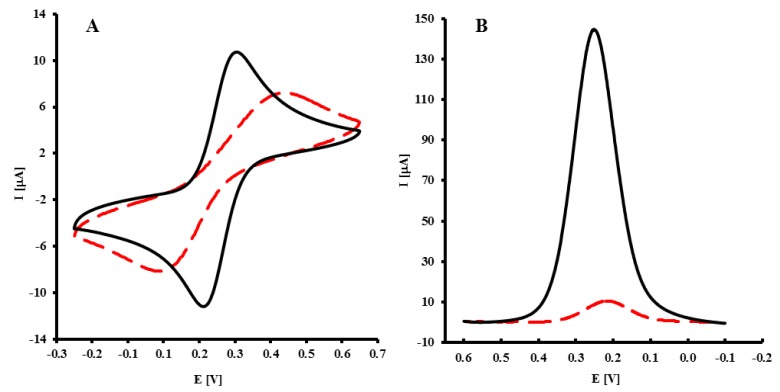
(**A**) Cyclic voltammograms (scan rate 0.1 V/s) and (**B**) square wave voltammograms (frequency 25 Hz) of: bare GCE (black curve); GCE-CO-NH-ssDNA modified electrode (dashed curve); Solution composition: 1 mM K_3_[Fe(CN)_6_]/ K_4_[Fe(CN)_6_] in 0.1 M PBS buffer, pH 7.4. The measuring conditions: three electrode configurations: GC working electrode, Ag/AgCl reference electrode and Pt counter electrode.

The immobilization of NH_2_-ssDNA probe was confirmed by CV and OSWV in the presence of Fe(CN)_6_^3−/4−^ as a redox marker. As expected in the CV, the redox marker showed reversible behavior on a bare GC electrode, with a peak-to-peak separation ΔE_p_ = 69 ± 2 mV (Figure 2A, black curve). After the covalent attachment of the NH_2_-ssDNA probe on the GCE, the peak current decreased, while peak-to-peak separation increased to 297 ± 15 mV (Figure 2A, dashed curve). This indicated a decreased in the reversibility of the system. In OSWV, for a bare GC electrode, the peak potential E = 256 ± 9 mV, and peak current I = 136 ± 10 μA (Figure 2B, black curve) were recorded. After immobilization of the NH_2_-ssDNA probe, the peak current decreased to I = 10 ± 1 μA, and the peak potential shifted to E = 206 ± 7 mV (Figure 2B, dashed curve). The results obtained by OSWV are in good accordance with CV measurements.

### 3.2. Determination of PPV Specific DNA Sequences with the Electrochemical Genosensor

The presented genosensor was applied for screening of the interactions between the ssDNA probe attached to the electrode surface and different ssDNA targets present in sample solution. The hybridization reactions were monitored by OSWV. This technique is more sensitive than CV, because the current is measured at the end of each potential change, right before the next, so that the contribution to the current signal from the capacitive charging current is diminished [47].

**Figure 3.**
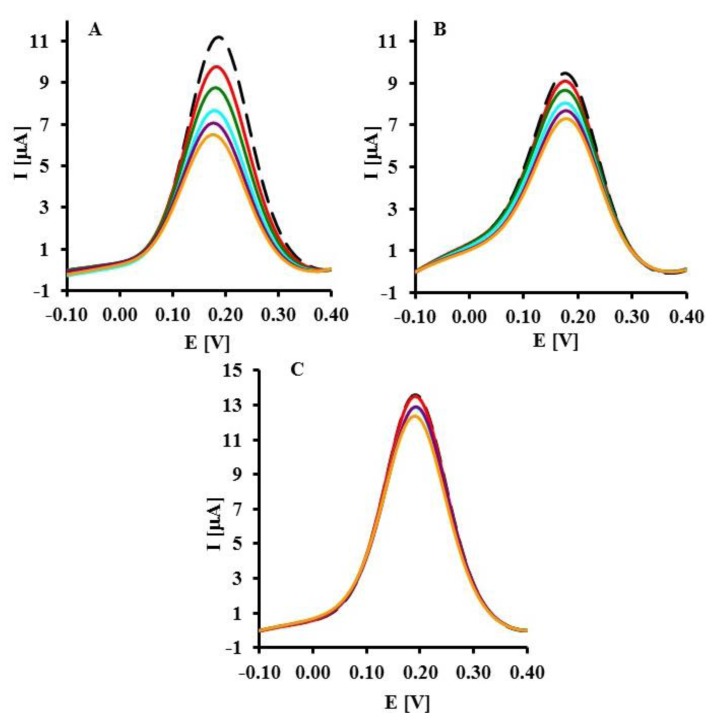
Typical OSWV curves obtained for electrodes modified with GCE-CO-NH-ssDNA-EA upon hybridization with DNA targets. Black curve—before hybridization and the following curves upon hybridization with targets: (**A**) LC, (**B**) SC and (**C**) PC at concentration range: 1–8 [pM]. Solution composition: 1 mM [Fe(CN)_6_]^3−/4−^ in 0.1 M PBS (pH = 7.4). The measuring conditions: three electrode configurations: GCE-CO-NH-ssDNA modified electrode, Ag/AgCl reference electrode and Pt counter electrode, frequency 25 Hz.

**Figure 4.**
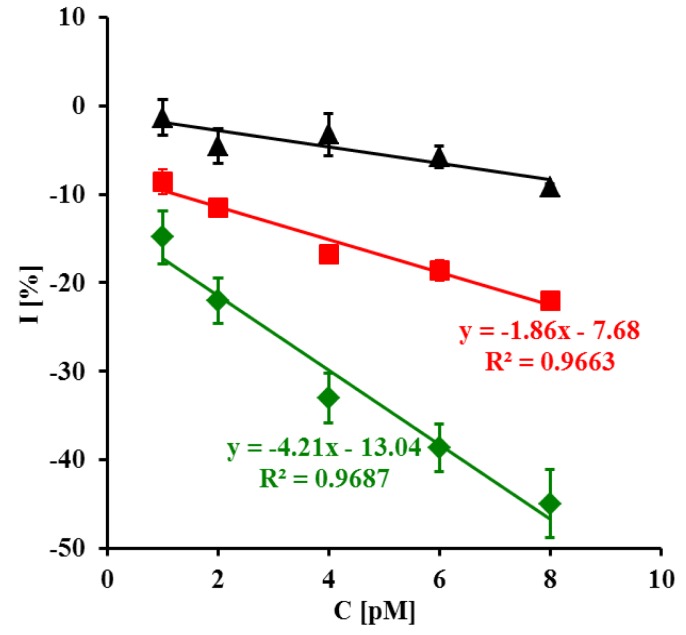
The relationship of I = (I_n_ – I_0_)/I_0_ (%) *vs.* log C target [pM] of ( 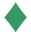) LC, ( 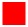) SC and ((▲)) PC. I_n_ is the value of peak current after detection of given target concentration, and I_0_ is the value of peak current without presence of target in pure 0.1 M PBS, pH 7.4 (n = 5/6).

The representative square wave voltammograms recorded in the presence of LC, SC complementary sequences and 20-mer PC sequence of oligonucleotides are presented in Figure 3. Upon increasing the concentration of LC and SC targets, a decrease of peak current was observed (Figure 3A,B). The highest concentration of LC and SC targets (8 pM) caused 42.7 ± 3.8% and 22.0 ± 0.8% (n = 6) decreasing of peak current, respectively. In the presence of 8 pM of 20-mer partially complementary DNA sequences with four complementary bases (PC) only 9.21 ± 0.34% peak current decreasing was observed (Figure 4).

Limits of detection (LOD) were calculated based on the standard deviation of the response and the slope of the calibration curve:

(1) LOD=3.3σ/S

where σ is the standard deviation of the response and S is the slope of the calibration curve [48]. The detection limit for the SC and LC was 2.4 pM (31.0 pg/mL) (S/N = 3) and 2.3 pM (29.5 pg/mL) (S/N = 3), respectively. In the case of 42-mer oligonucleotide sequence, we obtained a larger standard deviation in comparison to 22-mer oligonucleotide sequence. Therefore, in spite of larger response of 42-mer oligonucleotide sequence, the detection limits of both targets were similar.

In the next step, the genosensor was applied for detection of the specific DNA sequences derived from PPV in the extracts prepared from healthy (C^−^ PPV) and infected plants (C^+^ PPV). C^+^ PPV DNA sequence derived from the plant extract is a sequence complementary to a conservative region of the virus genome, located at the 3′ end. Depending on the virus isolate, the exact length (number of nucleotides) might be different. Therefore, in the case of plant extracts, the concentration of DNA was expressed in [pg/mL].

**Figure 5.**
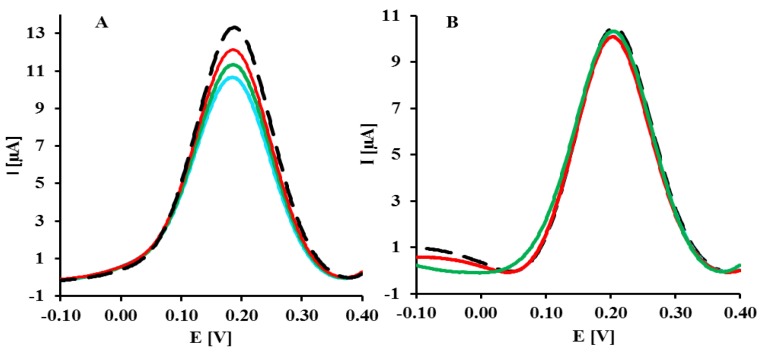
Typical OSWV curves obtained for electrodes modified with GCE-CO-NH-ssDNA-EA upon hybridization with DNA samples from plant extracts. Dashed curve—before hybridization and the following curves upon hybridization with target (**A**) C^+^ PPV and (**B**) C^−^ PPV at the concentrations: 10, 30 and 50 pg/mL. Solution composition: 1 mM [Fe(CN)_6_]^3−/4−^ in 0.1 M PBS (pH = 7.4). The measurement conditions: three electrode configurations: GCE-CO-NH-ssDNA modified electrode, Ag/AgCl reference electrode and Pt counter electrode, frequency 25 Hz.

**Figure 6.**
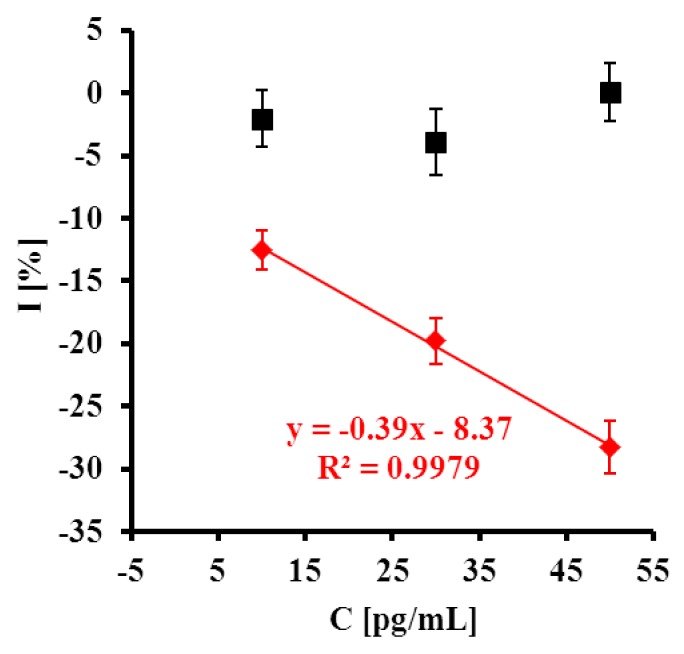
The relationship of I = (I_n_–I_0_)/I_0_ (%) *vs.* log C target [pg/mL] of ( 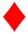) C^+^ and (■) C^-^. I_n_ is the value of peak current after detection of given concentration of target, and I_0_ is the value of peak current without presence of target in pure 0.1 M PBS pH 7.4 (n = 5/6).

Upon increasing the content of C^+^ PPV, a decrease of peak current was observed (Figure 5A). In a case of C^−^ PPV the response was negligible (Figure 5B). The highest concentration of C^+^ PPV target (50 pg/mL) caused a 28.3 ± 2.1% decrease in peak current. In the presence of C^−^ PPV target the peak current remained almost constant (0.07 ± 2.3%) (Figure 6). The detection limit for the C^+^ PPV target was 12.8 pg/mL.

At present, PPV is mostly assayed using an approved serological technique—enzyme-linked immunosorbent assay (ELISA) [49–51]. The sensitivity of this technique is worse (in ng/mL range) in comparison to th the genosensor presented (in pg/mL range). Highly sensitive reverse transcription polymerase chain reaction (RT-PCR) based assays are becoming more popular [7,52,53]. Unfortunately this device is costly and requires labor-intensive sample preparation. Electrochemical biosensors are a very promising alternative to the currently used traditional methods. They are cost-effective analytical devices, which demand very small sample volume (in μL level) and offer fast and simple analysis with the possibility of miniaturization. In addition, they are not sensitive to matrix components. In this paper, DNA sequences present in the plant extract were determined with the detection limit of 12.8 pg/mL. A similar sensitivity (10 pg/mL) was obtained for determination of PPV virus by an electrochemical immunosensor developed by our group [54].

To our knowledge, there are no reports about the determination of specific sequences of PPV using genosensors, especially in plant extracts. Until now, only the successful application of DNA biosensor based on differential pulse anodic stripping voltammetry for detection of plant pathogen—cauliflower mosaic virus 35 S gene sequences—with a detection limit of 4.38 pM has been reported [55]. The proposed genosensor displays two times better sensitivity for LC (2.3 pM) and SC (2.4 pM) targets, than that published by Sun and co-workers [55].

## 4. Conclusions/Outlook

The glassy carbon electrode (GCE) modified with NH_2_-ssDNA probe characterized by CV and OSWV were used for the voltammetric determination of specific ssDNA sequences derived from PPV virus present in buffer and plant extracts. The electrochemical genosensor was able to detect specific ssDNA sequences characterized for PPV virus. The similar detection limit 2.4 pM (31.0 pg/mL) and 2.3 pM (29.5 pg/mL) in the concentration range 1–8 pM were observed in the presence of the 22-mer (SC) and 42-mer (LC) sequences of PPV, respectively. The genosensor was very selective. The 20-mer partially complementary DNA sequences with four complementary bases (PC) generated a weak response. In the case of sequences derived from plant extract infected with PPV (C^+^ PPV) the detection limit was 12.8 pg/mL. In addition, the extract from healthy plant (C^−^ PPV) gave no response. This proved that the genosensor presented is free of plant matrix effect.

Good sensitivity and selectivity, demanding of small sample volume and simple fabrication, with the opportunity of miniaturization justify the proposed genosensor application for determination of specific DNA sequences derived from PPV virus present plant extracts.

## References

[1] Sochor J., Babula P., Adam V., Krska B., Kizek R. (2012). Sharka: The past, the present and the future. Viruses.

[2] López-Moya J.J., Fernández-Fernández M.R., Cambra M., García J.A. (2000). Biotechnological aspects of plum pox virus. J. Biotech..

[3] James D., Thompson D. (2006). Hosts and symptoms of Plum pox virus: ornamental and wild Prunus species. Bull. OEPP.

[4] Adams A.N., Guise C.M., Crossley S.J. (1999). Plum pox virus detection in dormant plum trees by PCR and ELISA. Plant Pathol..

[5] Thomidis T., Karajiannis I. (2003). Using ELISA and PCR to test the potential for spread of plum pox virus by seeds of different stone fruit cultivars. N. Z. J. Crop Hortic. Sci..

[6] Dovas C.I., Mamolos A.P., Katis N.I. (2002). Fluctuations in concentration of two potyviruses in garlic during the growing period and sampling conditions for reliable detection by ELISA. Ann. Appl. Biol..

[7] Schneider W.L., Herman D.J., Stone A.L., Damsteegt V.D., Frederick R.D. (2004). Specific and quantification of Plum pox virus by real-time fluorescent reverse transcription—PCR. J. Virol. Methods.

[8] Mavrič Pleško I., Viršček Marn M., Toplak N. (2011). Total RNA extraction method and *Prunus* species influence the detection of plum pox potyvirus by real-time RT-PCR. Acta Agric. Slov..

[9] Perumal V., Haskim U. (2014). Advances in biosensors: Principle, architecture and applications. J. Appl. Biomed..

[10] Singh R., Mukherjee M.D., Sumana G., Gupta R.K., Sood S., Malhotra B.D. (2014). Biosensors for pathogen detection: A smart approach towards clinical diagnosis. Sens. Actuators B Chem..

[11] Guth U., Vonau W., Zosel J. (2009). Recent developments in electrochemical sensor application and technology—A review. Meas. Sci. Technol..

[12] Perdikaris A., Vassilakos N., Yiakoumettisa I., Kektsidou O., Kintzios S. (2011). Development of a portable, high throughput biosensor system for rapid plant virus detection. J. Virol. Methods.

[13] Wang X., Lu X., Chen J. (2014). Development of biosensor technologies for analysis of environmental contaminants. TrEAC Trends Environ. Anal. Chem..

[14] Yáñez-Sedeño P., Agüí L., Villalonga R., Pingarrón J.M. (2014). Biosensors in forensic analysis. A review. Anal. Chim. Acta.

[15] Pienno P.A.E., Krull U.J. (2005). Trends in the development of nucleic acid biosensors for medical diagnostics. Anal. Bioanal. Chem..

[16] Paleček E., Bartosik M. (2012). Electrochemistry of nucleic acids. Chem. Rev..

[17] Paleček E. (2002). Past, present and future of nucleic acids electrochemistry. Talanta.

[18] Vercoutere W., Akeson M. (2002). Biosensors for DNA sequence detection. Curr. Opin. Chem. Biol..

[19] Berney H., West J., Haefele E., Alderman J., Lane W., Collins J.K. (2000). A DNA diagnostic biosensor: Development, characterisation and performance. Sens. Actuators B Chem..

[20] Zhu D., Liu J., Tang Y., Xing D. (2010). A reusable DNA biosensor for the detection of genetically modified organism using magnetic bead-based electrochemiluminescence. Sens. Actuators B Chem..

[21] Lee J.-G., Yun K., Lim G.-S., Lee S.E., Kim S., Park J.-K. (2007). DNA biosensor based on the electrochemiluminescence of Ru(bpy)_3_^2+^ with DNA-binding intercalators. Bioelectrochemistry.

[22] Shi Y., Huang W.T., Luo H.Q., Li N.B. (2011). A label-free DNA reduced graphene oxide-based fluorescent sensor for highly sensitive and selective detection of hemin. Chem. Commun..

[23] Qiu S., Li X., Xiong W., Xie L., Guo L., Lin Z., Qiu B., Chen G. (2013). A novel fluorescent sensor for mutational p53 DNA sequence detection based on click chemistry. Biosens. Bioelectron..

[24] Kleo K., Kapp A., Ascher L., Lisdat F. (2011). Detection of vaccinia virus DNA by quartz crystal microbalance. Anal. Biochem..

[25] Prakrankamanant P., Leelayuwat C., Promptmas C., Limpaiboon T., Wanram S., Prasongdee P., Pientong C., Daduang J., Jearanaikoon P. (2013). The development of DNA-based quartz crystal microbalance integrated with isothermal DNA amplification system for human papillomavirus type 58 detection. Biosens. Bioelectron..

[26] Xue T., Cui X., Guan W., Wang Q., Liu Ch., Wang H., Qi K., Singh D.J., Zheng W. (2014). Surface plasmon resonance technique for directly probing the interaction of DNA and graphene oxide and ultra-sensitive biosensing. Biosens. Bioelectron..

[27] Li Y., Yan Y., Lei Y., Zhao D., Yuan T., Zhang D., Cheng W., Ding S. (2014). Surface plasmon resonance biosensor for label-free and highly sensitive detection of point mutation using polymerization extension reaction. Colloids Surf. B.

[28] Stobiecka M., Cieśla J.M., Janowska B., Tudek B., Radecka H. (2007). Piezoelectric sensor for determination of genetically modified Soybean Roundup Ready^®^ in samples not amplified by PCR. Sensors.

[29] Lucarelli F., Tombelli S., Minunni M., Marazza G. (2008). Electrochemical and piezoelectric DNA biosensors for hybridisation detection. Anal. Chim. Acta.

[30] Kirimli C.E., Shih W.H., Shih W.Y. (2014). DNA hybridization detection with 100 zM sensitivity using piezoelectric plate sensors with an improved noise-reduction algorithm. Analyst.

[31] Drummond T.G., Hill M.G., Barton J.K. (2003). Electrochemical DNA sensors. Nat. Biotechnol..

[32] Malecka K., Grabowska I., Radecki J., Stachyra A., Góra-Sochacka A., Sirko A., Radecka H. (2012). Voltammetric detection of a specific DNA sequence of Avian Influenza Virus H5N1 using HS-ssDNA probe deposited onto gold electrode. Electroanalysis.

[33] Malecka K., Grabowska I., Radecki J., Stachyra A., Góra-Sochacka A., Sirko A., Radecka H. (2013). Electrochemical detection of avian influenza virus genotype using amino-ssDNA probe modified gold electrodes. Electroanalysis.

[34] Cui H.-F., Cheng L., Zhang J., Liu R., Zhang Ch., Fan H. (2014). An electrochemical DNA sensor for sequence-specific DNA recognization in a homogeneous solution. Biosens. Bioelectron..

[35] Zheng J., Chen C., Wang X., Zhang F., He P. (2014). A sequence-specific DNA sensor for Hepatitis B virus diagnostics based on the host–guest recognition. Sens. Actuators B Chem..

[36] Batchelor-McAuley C., Wildgoose G.G., Compton R.G. (2009). The physicochemical aspects of DNA sensing using electrochemical methods. Biosens. Bioelectron..

[37] Liu A., Wang K., Weng S., Lei Y., Lin L., Chen W., Lin X., Chen Y. (2012). Development of electrochemical DNA biosensors. TrAC Trends Anal. Chem..

[38] Gang L., Ying W., Zi-Ying Z., Shu-Zhen R., Chun-Hai F. (2011). Deoxyribonucleic acid molecular design for electrochemical biosensors. Fenxi Huaxue.

[39] Ivnitski D., O’Neil D.J., Gattuso A., Schlicht R., Calidonna M., Fisher R. (2003). Nucleic acid approaches for detection and identification of biological warfare and infectious disease agents. BioTechniques.

[40] Wang J. (2002). Electrochemical nucleic acid biosensors. Anal. Chim. Acta.

[41] Fojta M. (2002). Electrochemical sensors for DNA interactions and damage. Electroanalysis.

[42] Chang S., Puryear J., Cairney J. (1993). A simple and efficient method for isolating RNA from pine trees. Plant Mol. Biol. Rep..

[43] Pan T., Xiao Z.-D., Huang P.-M. (2009). Characterize the interaction between polyethylenimine and serum albumin using surface plasmon resonance and fluorescence method. J. Lumin..

[44] Patel N., Davies M.C., Heaton R.J., Roberts C.J., Tendler S.J.B., Williams P.M. (1998). A scanning probe microscopy study of the physisorption and chemisorption of protein molecules onto carboxylate terminated self-assembled monolayers. Appl. Phys. A.

[45] Umezawa Y., Aoki H. (2004). Ion channel sensors based on artificial receptors. Anal. Chem..

[46] Pang D.-W., Zhang M., Wang Z.-L., Qi Y.-P., Cheng J.-K., Liu Z.-Y. (1996). Modification of glassy carbon electrode and electrodes with DNA. J. Electroanal. Chem..

[47] Bard J.A., Faulkner L.R. (2001). Electrochemical Methods.

[48] Swartz M.E., Krull I.S. (2012). Handbook of Analytical Validation.

[49] Byzova N.A, Safenkova I.V., Chirkov S.N., Avdienko V.G., Guseva A.N., Mitrofanova I.V., Zherdev A.V., Dzantiev B.B., Atabekov J.G. (2010). Interaction of plum pox virus with specific colloidal gold-labeled antibodies and development of immunochromatographic assay of the virus. Biochemistry.

[50] Croft H., Malinowski T., Krizbai L., Mikec I., Kajic V., Reed C., Varga A., James D. (2008). Use of Luminex xMAP-derived Bio-Plex bead-based suspension array for specific detection of PPV W and characterization of epitopes on the coat protein of the virus. J. Virol. Methods.

[51] Rubio M., Ruiz D., Egea J., Martínez-Goméz P., Dicenta F. (2008). Evaluation of apricot resistance to Plum pox virus (Sharka) in controlled greenhouse and natural field conditions. Scientia Hort..

[52] Olmos A., Bertolini E., Cambra M. (2007). Isothermal amplification coupled with rapid flow-through hybridisation for sensitive diagnosis of Plum pox virus. J. Virol. Methods.

[53] Zhang S., Ravelonandro M., Russell P., McOwen N., Briard P., Bohannon S., Vrient A. (2014). Rapid diagnostic detection of plum pox virus in Prunus plants by isothermal AmplifyRP^®^ using reverse transcription—Recombinase polymerase amplification. J. Virol. Methods.

[54] Jarocka U., Wąsowicz M., Radecka H., Malinowski T., Michalczuk L., Radecki J. (2011). Impedimetric immunosensor for detection of Plum Pox Virus in plant extracts. Electroanalysis.

[55] Sun W., Zhong J., Qin P., Jiao K. (2008). Electrochemical biosensor for the detection of cauliflower mosaic virus 35 S gene sequences using lead sulfide nanoparticles as oligonucleotide labels. Anal. Biochem..

